# From Pregnancy Pains to Paralysis: An Erroneous Intrathecal Digoxin Administration Case Report and Review of Medical Errors

**DOI:** 10.7759/cureus.64764

**Published:** 2024-07-17

**Authors:** Brianna M Holcomb, Saumya Shah, Rahul Shah

**Affiliations:** 1 Emergency Medicine, California Health Sciences University College of Osteopathic Medicine, Clovis, USA; 2 Anesthesiology, California Health Sciences University College of Osteopathic Medicine, Clovis, USA; 3 Neurology/Neuro Critical Care, Ochsner/Louisiana State University Health Shreveport, Shreveport, USA

**Keywords:** medication error in pregnancy, case report, intrathecal digoxin, drug administration error, medical error

## Abstract

Digoxin is a Na-K ATPase inhibitor commonly used to treat heart failure and atrial fibrillation. It is only approved for oral or intravenous (IV) use. There is no approved indication for intrathecal administration. Only four previously reported cases of intrathecal digoxin administration in pregnant patients are in the literature. We present a patient who had an unfortunate case of erroneous intrathecal Digoxin administration following an elective Cesarean section. Post-delivery, the patient's mental status deteriorated. She became unresponsive and remained comatose for 11 days. Brain magnetic resonance imaging (MRI) showed diffuse, patchy hyperintensities involving bilateral frontotemporal lobes and basal ganglia. A spine MRI showed extensive cervical and thoracic cord edema. At discharge, the patient was paraplegic with no sensation or motor response below the level of T10. At the 90-day follow-up, she had intact mental status and minimal improvement in motor strength and sensation below T10 and was reportedly breastfeeding. This is an unfortunate case of severe neurological deficits resulting from a grave medical error, which continues to be a prevalent issue in the United States healthcare system.

## Introduction

An estimated 5% of adult inpatients experience medical errors yearly, with anywhere from 98,000 to 440,000 of those errors resulting in fatalities [1-5]. Approximately 8.9% of medical errors are due to adverse drug events (ADEs). Although some errors involving medications can be benign, certain medications and routes of administration can lead to severe adverse events, as was the case for our patient [6].

Digoxin is a commonly used drug for cardiovascular disorders, including arrhythmias and heart failure. Oral and intravenous (IV) administration are the only approved forms of administration. Its pharmacokinetics do not indicate intrathecal administration [7-8]. Digoxin reversibly binds to the alpha subunit of Na/K-ATPase on the membranes of all cells, effectively inhibiting the protein's function. Although this can be useful in cardiac rhythm control and contractility of the heart, inhibition of this protein can also significantly impact the function of neurons, potentially leading to severe neurologic or psychiatric symptoms [9]. Several studies have examined the clinical manifestations of acute and chronic digoxin toxicity, including ocular, gastrointestinal, cardiac, and, in our interests, neuropsychiatric symptoms. Since digoxin has no approved indication for intrathecal use, little is known about its direct effects on the central nervous system (CNS) when injected into the intrathecal space. Even less is known regarding appropriate management options in the rare cases where this has occurred.

We describe a case of accidental intrathecal administration of digoxin, which occurred during an elective cesarean section and led to severe neurological deficits. There are only four prior cases of adverse effects from erroneous epidural digoxin administration in pregnant patients in the literature [7-8,10-11]. The progression and management of the complications during hospitalization and at 1-week, 6-week, and 12-week post-discharge follow-up visits are discussed.

## Case presentation

**Figure 1:**
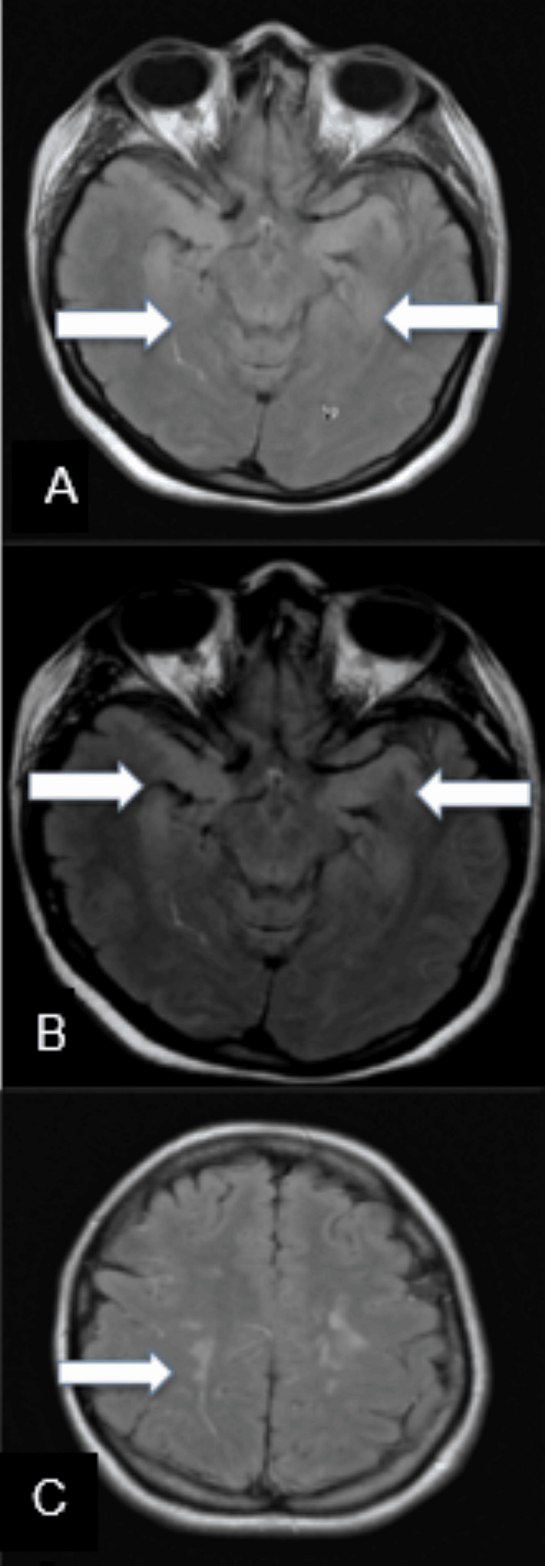
Axial MR FLAIR images of the brain. There is extensive T2/FLAIR hyperintensity of the limbic structures including the hippocampi (A), and temporal amygdalae and para hippocampal gyri (B), as well as the bilateral supratentorial deep white matter (C).

A 34-year-old gravida 3, para 2 Hispanic female underwent elective cesarean section with two separate attempts at regional spinal anesthesia with bupivacaine. The first injection failed to achieve adequate anesthesia. Two hours after delivering a healthy child, the patient developed altered mental status and rapidly became unresponsive. She had three generalized tonic-clonic seizures, was emergently intubated for airway protection, and received levetiracetam. At 24 hours, the patient remained comatose despite the cessation of seizures. A continuous electroencephalogram (EEG) revealed no epileptic discharges or electrographic seizures. Magnetic resonance imaging (MRI) of the brain showed diffuse, patchy hyperintensities involving the gray matter in bilateral temporal lobes, bilateral insular cortices, bilateral frontal lobes, and bilateral basal ganglia (Figure 1).

**Figure 2:**
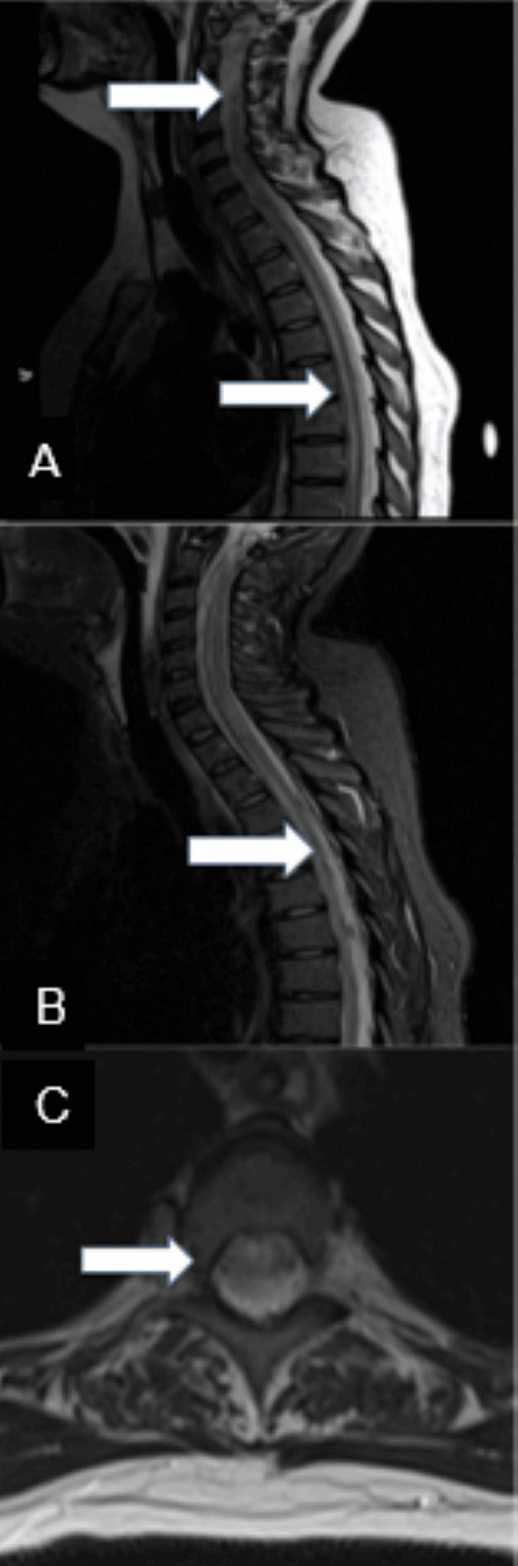
Sagittal T2 weighted (A), and STIR (B), and axial T2 weighted (C) images of the cervical and thoracic spine are shown. The arrows indicate the extensive areas of edema throughout the spinal cord.

Risk management discovered that the patient had erroneously received digoxin instead of bupivacaine as the initial injection during the C-section. Therapeutic serum levels of digoxin confirmed this finding. She was immediately transferred to the neuro ICU at our hospital. The MRI spine showed extensive cervical and thoracic cord edema (Figure 2). Cerebrospinal fluid (CSF) analysis showed 476 white blood cells (WBC), a protein count of 211, and a lactic acid level of 7.6, indicating a severe CNS inflammatory response. The patient was started on broad-spectrum antibiotics, which were duly discontinued once all infective workups were negative. She received high-dose methylprednisolone, 1000 mg IV daily for five doses, with some subsequent clinical and neurological improvement. A neurological examination one week into admission was significant for occasional spontaneous eye-opening and bilateral upper extremity withdrawal but not following commands. Serum digoxin levels were undetectable at this point, so the decision was made not to give digoxin Fab. Due to a lack of significant clinical improvement, she was started on a five-day course of IVIG. The patient tolerated IVIG well without any complications. Her mental status continued to show improvement. Eleven days post-initial ictus, she was extubated and cleared for oral diet three days later. CSF analysis showed resolution with only nine WBCs, normal protein, and lactic acid. An MRI of the spine showed complete resolution except for some residual cord edema in the thoracic cord. The neurological examination was significant for 5/5 strength in bilateral upper extremities, intact speech, poor recall, following commands with slow response time, and no sensation or motor response below the T10 level.

At the time of discharge, around 14 days after being transferred to the neuro ICU at our hospital, she could sit up, have conversations, hold her newborn baby, and breastfeed. She had lost all memory of her pregnancy and continued to be paraplegic. She was discharged from the hospital the next day with continued outpatient rehabilitation. Six weeks post-discharge, the patient was ambulating in a wheelchair, and ten weeks post-discharge, she took her first steps without the zero-gravity assist device. At the 90-day follow-up, she was nursing her baby, and a neurological examination revealed intact mental status with 3/5 strength in bilateral lower extremities and some sensation to light touch and pain.

## Discussion

In this case, the significant medical error involved the accidental administration of digoxin instead of bupivacaine during the regional spinal anesthesia attempts. This error occurred due to its placement in close proximity to other medications in the pyxis and the anesthesiologist's failure to scan the medication prior to administration. The anesthesiologist's failure to report the error immediately due to a fear of consequences further delayed the diagnosis and appropriate management attempts. Digoxin inhibits the sodium-potassium ATPase pump, altering ion balances across cell membranes, particularly in cardiac cells. This leads to increased intracellular calcium levels, positively affecting cardiac contractility and output. However, if administered intrathecally, this disruption of normal ion gradients could theoretically lead to severe neurological complications.

The therapeutic window of digoxin is relatively narrow, meaning that even slight deviations from the appropriate dosage can result in toxic effects. Digoxin targets cardiac cells in therapeutic doses, but when administered in excess, it can lead to serious neurological consequences. A literature review has shown intrathecal digoxin to have the most devastating consequences of any erroneously administered epidural or intrathecal cardiac drug. It has been postulated that when digoxin is administered intrathecally, it disrupts the ion gradients and calcium signaling in neurons, affecting cell signal propagation. Inappropriately elevated intracellular calcium levels disrupt normal neuronal signaling, potentially leading to hyperexcitability, oxidative stress, inflammation, and cell damage. This disruption in calcium homeostasis theoretically causes a wide range of neurological deficits, including altered mental status, seizures, and paralysis. It is important to note that in each of the reported cases of intrathecal administration, the serum levels of digoxin were shown to be within the therapeutic window, meaning that neurologic symptoms of encephalopathy, seizures, confusion, and fatigue would more likely be due to direct neurological cell damage rather than systemic toxicity [12].

Common imaging findings of intrathecal digoxin administration include ischemia, demyelination, myelitis, and edema [12]. In this case, the patchy hyperintensities observed in the brain MRI were indicative of widespread inflammation and ischemia in various brain regions, including the temporal lobes, insular cortices, frontal lobes, and basal ganglia. These changes could explain the patient's altered mental status, seizures, and subsequent paraplegia.

Although formal treatment protocols have not been put in place due to the rarity of their occurrence, historical treatment procedures included digoxin-immune antibodies and hemodialysis, steroids, antiepileptics, and mechanical ventilation when indicated. There is discussion as to whether early CSF lavage could theoretically prevent a severe degree of damage due to the delayed onset of the medication, but no conclusions have been drawn [12]. The patient's improvement over time was likely a result of the aggressive immunomodulatory treatment and the gradual resolution of inflammation and edema in the central nervous system. However, memory loss and residual neurological deficits, including persistent paraparesis, highlight the severe and lasting consequences of the initial medical error involving Digoxin administration.

Healthcare facilities in the United States continue to struggle with minimizing the rate of medical errors. Although it is difficult to report an accurate rate of annual medical errors in the United States due to a lack of reporting and biases, many studies estimate that 4.8-5.3% of adult inpatients experience medical errors annually, impacting an average of seven million individuals [2,4,5,13]. In 2000, a study estimated the resulting fatalities to be 98,000, with more recent studies showing an alarming range of 250,000 to 440,000 lives claimed annually [1-3]. Sunshine et al. published a study in 2019 that reported that 8.9% of these fatal medical errors were due to ADE [6]. The most common causes largely involve drug administration: transcription errors, timing of administration, dosage mistakes (incorrect, omitted, or doubled dosages), look-alike or sound-alike drugs being administered instead, different medications being stored in the same place or pulled from the same drawer, misconnection of lines, communication errors (including interruptions) during administration, and a lack of training or experience of involved personnel [4-6,12-20].

The risk of adverse outcomes due to inappropriate administration of medications seems to result in more devastating outcomes for drugs given through neuraxial routes. This is of particular concern in operating room settings where cardiac medications are regularly stored alongside others; vasopressors are often prepared ahead of time; vials have been found to be left on the anesthesia platform and forgotten; and patients with multiple lines present organization issues with keeping lines and inputs straight while in a hectic environment [16].

One primary concern is that the factors contributing to these errors seem to be persistent. Recognizing the severity of the situation, many states and institutions have implemented changes over the past several years to mitigate medical errors and ADEs. The use of EMRs alongside computerized physician order entry systems (CPOEs) and clinical decision support systems has helped to decrease medication errors while the medications are still being ordered [3,14]. Certain states have adopted electronic surveillance, medication error screening, and error reporting systems, which studies have shown to be effective [3,4]. These systems allow for analyzing the source of the error, timely warning and treatment when errors occur, and planning for further prevention of similar errors [3,7].

Additional efforts have been made to improve procedures and instrumentation. Institutions have utilized the total capacity of their healthcare teams by having clinical pharmacists present during rounds to review medication orders [3]. Medications are now barcoded, and non-Luer lock lines have been put in place [14]. Although the universality of the Luer lock line is convenient, it has also enabled medication errors since its inception. In the aftermath of erroneous drug administration errors, more specific lines (such as the NR-Fit for neuraxial and regional anesthesia catheters) have been designed and put into practice [14,17,19]. A team at the University of Iowa has designed a multitype syringe port that would allow only specific categories of drugs to be locked into an IV line-one port for each drug class [13].

One of the significant obstacles to improvement is that discussions about errors only occur in limited and confidential forums [2]. Certain healthcare specialties hesitate to report errors due to fear of accusations, and some institutions are not improving their response following error reports [13]. There is speculation that this may be due to an overreliance on the malpractice system and a lack of incentive to invest in and improve patient safety [3]. However, we need to understand the problem better to solve it [2]. Individuals should be encouraged to report errors. Regular error analysis should be run with a focus on root-cause analysis to ensure that each situation is used for learning and improvement.

An error like accidental intrathecal digoxin administration necessitates considering further ways to decrease medication errors to avoid catastrophic consequences. Changes must occur at every level of the institution. Labels for look-alike and sound-alike medications should be differentiated further, and storage of these medications should be separated. Barcoding should be utilized and enforced in institutional policy. Personnel training should be consistent and continual, and healthcare colleagues should continue to double-check medication administrations and hold each other accountable to institutional policies. Reasons for error should be discussed, and error analysis should be performed with the intent to follow through on necessary changes.

## Conclusions

Our case demonstrates that drug administration errors persist despite advances in electronic medical records and automated medication dispensing systems, and the problem involves multiple factors. Frequently, the consequences of these errors are severe and catastrophic. Healthcare organizations and institutions need to continue investing in improving medication delivery systems. Additionally, creating a more transparent means of error reporting and discussion is vital. If these issues are not addressed or discussed, they are unlikely to be fixed. Just like there are forums to discuss medical advances in the field, there need to be more forums at institutional, state, and national levels to discuss these errors in a guilt-free and litigation-free environment to use the collective skill set in finding solutions to a problem that has as high a medical and economic impact as any other medical condition.
